# Prevalence and factors associated with pre-hospital delay among acute stroke patients at Mulago and Kiruddu national referral hospitals, Kampala: a cross-sectional study

**DOI:** 10.1186/s12883-023-03413-1

**Published:** 2023-10-21

**Authors:** Keith Twirire Kakame, Jane Nakibuuka, Nelson Mukiza, Irene Andia-Biraro, Mark Kaddumukasa, Chris Burant, Elly Katabira, Martha Sajatovic

**Affiliations:** 1 Department of Medicine, College of Health Sciences, Makerere University, P.O Box 7072, Kampala, Uganda; 2 Mulago National Referral Hospital, P.O Box 7051, Kampala, Uganda; 3 RhineCynth Advisory Limited, Kampala, Uganda; 4 Frances Payne Bolton School of Nursing, Case Western Reserve University, 10900 Euclid Ave, Cleveland, OH, TOMO USA; 5 Neurological and Behavioral Outcomes Center, University Hospitals Cleveland Medical Center & Case Western Reserve University School of Medicine, 11100 Euclid Avenue, Cleveland, OH 44106 USA

**Keywords:** Pre-hospital delay, Stroke, Acute stroke care, Low and middle-income countries

## Abstract

**Background:**

Despite advancements in acute stroke care, acute stroke patients present late for care resulting in high mortality and poor functional outcomes. This study determined the prevalence of pre-hospital delay and associated factors among adult acute stroke patients in Uganda.

**Methods:**

In a hospital based, cross-sectional study, one hundred and forty-three study participants with confirmed acute stroke presenting to the emergency units of Mulago and Kiruddu national referral hospitals were enrolled. Using an interviewer-administered questionnaire, details on sociodemographics, onset of stroke, arrival at the tertiary facility, health system and clinical factors were collected. Descriptive statistics and modified Poisson regression analyses were performed to determine factors associated with prehospital delay.

**Results:**

Among the 143 study participants, nearly two-thirds (79/146) had ischemic stroke while a third (59/143) had haemorrhagic stroke. The mean age was 59 years (SD 16) and 51.7% of acute stroke patients were males. Ninety one percent (130/143) presented to the emergency unit after 3 hours. The majority (124/143) reported visiting lower-level facilities prior to referral to the tertiary facility. Staying outside Kampala district (PR: 1.28 (1.22–1.34), *p* < 0.001), and using hired or government ambulance for transport to tertiary facility (PR: 1.17 (1.13–1.20), *p* < 0.001) were associated with pre-hospital delay.

**Conclusions:**

Prevalence of pre-hospital delay among acute stroke patients presenting to public tertiary hospitals in Uganda is very high. The causes of pre hospital delay should be further explored qualitatively. Efforts to reduce prehospital delay should include improving pre-hospital transport systems for stroke patients.

## Introduction

The incidence of stroke has declined in high income countries (HIC) over the past two decades, while rising in low- and middle-income countries (LMICs) [1]. Worse still, LMICs have poor stroke outcomes compared to HIC with LMICs accounting for 87% stroke related deaths and disability adjusted life years globally [2].

The World Stroke Organisation through the Global Stroke Services Action Plan recommends early stroke assessment, diagnosis and management at health centres that can offer essential stroke services [3]. Early arrival of stroke patients allows for prompt evaluation of patients’ stroke subtypes and initiation of time-sensitive treatments such as intravenous fibrinolysis within 3 to 4.5 h of stroke symptoms for ischemic stroke and blood pressure control for haemorrhagic stroke [4].

Additionally, the American Stroke Association guidelines recommend that, in patients eligible for IV fibrinolysis, benefit of therapy is time dependent, and treatment should be initiated as quickly as possible [4]. As such, arriving at the hospital within 3 h after onset of stroke is usually considered beneficial for receiving timely treatment. Early arrival also allows for prevention and management of immediate complications of stroke such as aspiration pneumonia, fever, hypoglycaemia and increased intracranial pressure to minimize the impact of stroke and prevent further damage [3].

Late presentation to hospital after onset of stroke has been highlighted as one of the factors contributing to increased stroke morbidity and mortality [5]. Time to hospital presentation varies between high-income countries and low-income countries, across regions and across different health systems. The mean time to hospital presentation after onset of stroke has been reported to range from 1.2 to 98.8 h in a systematic review focusing on pre-hospital delay times [6].

Despite advances in acute stroke care, Uganda still has poor stroke outcomes with a high 30 day mortality ranging from 25–30% of patients admitted with stroke to tertiary facilities [7–9]. Late presentation of acute stroke patients could be contributing to the poor stroke outcomes observed among patients presenting to tertiary facilities –facilities that can offer Brain CT scan and other key essential stroke care services in Uganda. There is paucity of data on the prevalence of pre-hospital delay among acute stroke patients presenting to tertiary hospitals in Uganda. Additionally, factors associated with pre-hospital delay among acute stroke patients presenting to tertiary hospitals in Uganda are not known. 

Therefore, this study determined the prevalence of pre-hospital delay and associated factors among acute stroke patients presenting to Mulago and Kiruddu national referral hospitals in Uganda.

## Methods

### Study design and setting

This was a cross sectional study conducted between February and April 2022 at the emergency units of Mulago and Kiruddu national referral hospitals in Uganda. Mulago national referral hospital is the main public tertiary hospital in Kampala, located 4 km from Kampala city centre with a bed capacity of 1500 beds. It has an emergency unit, which receives all patients with suspected stroke. On average, this unit receives 45–50 patients with suspected stroke per month. Kiruddu national referral hospital is also a public tertiary hospital, located 13 km from Kampala city centre with a bed capacity of 200 beds. The hospital majorly serves adults with medical conditions and has an emergency unit, which receives all patients with suspected stroke. On average, this unit receives 30–35 patients with suspected stroke per month. At both hospitals, a medical officer or physician on duty initially reviews patients with suspected stroke before transfer to the neurology unit. In addition, each of the hospitals has a radiology unit with 24/7 computerised tomography (CT) scan services.

Each hospital has a 24/7 neurology unit headed by a neurologist where stroke patients receive comprehensive stroke care. The neurologist works with a team that includes physicians, medical officers, nursing officers and support staff. The hospitals do not have a dedicated stroke unit. The hospitals offer other auxiliary stroke services such as physiotherapy and ICU care. The hospitals do not currently offer fibrinolysis or thrombectomy services but the services are planned to be offered in the near future. The standard of care for acute stroke at Mulago and Kiruddu hospitals is a modification of the American Heart Association/American Stroke Association (AHA/ASA) guidelines. The hospitals’ catchment area includes Kampala district and surrounding districts. There is no clear national guidance on the stroke pathway and, as a result, the national referral hospitals receive both patients who come directly to the hospital after onset of stroke and patients referred from both public and private health facilities to access essential stroke care services.

### Study population

All adult patients with symptoms of stroke were screened and enrolled in the study if they met the inclusion criteria: 1) aged ≥ 18 years, 2) confirmed stroke with Brain CT scan, 3) presenting within 7 days of onset of stroke symptoms stroke to emergency units at Kiruddu and Mulago national referral hospitals in Uganda during the study period, and 4) provision of written informed consent to participate in the study. Patients were excluded if they were unconscious and with no legal guardian to provide key information and if the stroke occurred during inpatient hospitalisation. In addition, patients with missing variables were excluded.

### Sample size

To determine the prevalence of prehospital delay among acute stroke patients, the Kish (1965) formula for cross sectional studies was used [10]. The sample size was calculated assuming a prevalence of 90% of pre-hospital delay among the stroke patients presenting to a tertiary hospital as was found in a similar study in Nigeria [11]. Hence, using p (prevalence) = 0.90, q (complement of prevalence) = 0.10, d (acceptable degree of error) = 0.05, z (standard normal value corresponding to 95% confidence interval) = 1.96, and adjusting for a non-response of 5%, we calculated a sample size of 145.

To determine factors associated with pre-hospital delay among acute stroke patients, sample size was calculated using the formula below:

$$N=\frac{{\left[{Z}_{a}\sqrt{P(1-P)(1/{q}_{1}+1/{q}_{2})}+{Z}_{\beta }\sqrt{{P}_{1}\left(1-{P}_{1}\right)\left(1/{q}_{1}\right)+{P}_{2}(1-{P}_{2})(1/{q}_{2})}\right]}^{2}}{{\left({P}_{1}-{P}_{2}\right)}^{2}}$$

where Zα is the standard normal value corresponding to the level of significance (e.g., for a confidence level of 95%, α is 0.05 and the critical value is 1.96), Zβ is the standard normal value corresponding to the power of the study (e.g., for a power of 80%, β is 0.2 and the critical value is 0.84). Based on a study from Malawi, and considering readily available hospital fees as one of the factors associated with hospital arrival time after onset of symptoms [12], proportion of individuals with readily available fees (Group 1) with pre-hospital delay, (p1) = 0.65, proportion of individuals without readily available fees (Group 2) with pre-hospital delay (p2) = 0.93. Proportion of participants with readily available fees (group 1) (q1) = 0.476 and proportion of participants without readily available fees (group 2) (q2) = 0.524, and *P* = p1q1 + p2q2.

Based on the above formula, the calculated sample size was 68 participants per group, with a total sample size is 136 participants. Adjusting for an anticipated nonresponse rate of 5%, the calculated sample size for determining factors associated with prehospital delay was 143. Since the calculated sample size for determining prevalence of prehospital delay was slightly higher, we aimed to enrol 145 participants to determine the prevalence of prehospital delay and associated factors among acute stroke patients.

### Sampling procedure

All adults (aged ≥ 18 years) with symptoms of stroke within 7 days from onset, presenting to the emergency units, were screened and offered a Brain CT scan. A radiologist at each of the hospitals confirmed the diagnosis of stroke.

Once a diagnosis of stroke was confirmed, participants who consented to participate in the study were recruited consecutively, and a standardised questionnaire administered by the research team for those patients able to communicate. For patients not able to communicate, consent and information were obtained through the caregivers.

### Definitions

Prehospital delay was defined as the time interval from onset of symptoms of stroke to time of arrival at the tertiary hospital offering essential stroke services of more than 3 hours. Whereas it’s recommended to arrive at the appropriate hospital as early as possible, arriving at the hospital within 3 hours is usually considered beneficial for receiving timely treatment, as studied in previous studies [11–13]. Acute stroke referred to hospital presentation within 7 days since onset of stroke symptoms. Tertiary facility referred to a facility that could offer essential stroke services, particularly a brain CT scan, access to a physician with stroke expertise, and members of an interdisciplinary stroke team. The tertiary facilities in this study were Mulago national referral hospital (MNRH) and Kiruddu national referral hospital (KNRH).

### Data collection

A pre-tested and standardised questionnaire was used as a data collection tool. Depending on the language preference of the participant, the questionnaire was administered in either Luganda or English, the predominantly spoken languages in the catchment area. The principal investigator and trained research assistants administered the questionnaire to the participants. Information was collected on:

1. Time of onset of stroke symptom and time of arrival at the emergency unit of the tertiary hospital.
2. Socio-demographic factors i.e., age, sex, district of residence, highest level of education attained, employment status, religion, and marital status.
3. Symptom onset situational factors i.e., living alone or being alone at time of symptom onset, place of stroke- (home, work, other), presence of a bystander at time of stroke, first decision taken after stroke symptom onset- (consultation with a doctor, consultation with family member, consultation with a non-relative).
4. Health system factors i.e., distance from home to tertiary facility, availability of money for medical care, initial level of health facility visited, stroke related intervention at initial facility visited, type of transport means used to tertiary health facility.
5. Clinical factors i.e., stroke subtype, stroke severity, specific stroke symptoms, patient stroke history, stroke history among family members/acquaintance, presence of stroke risk factors such as Hypertension, Diabetes Mellitus and HIV, attending chronic care clinic.

Data collected were double entered into the computer using EPI-DATA version 3.1 software. Data were then backed up and archived in both soft and hard copies to avoid losses. To ensure confidentiality, code numbers instead of patients’ names were used. Questionnaires were stored in a locked cabinet for safety.

### Data analysis

Data were analysed using Stata version 14 (StataCorp LP, College Station, TX, USA). Descriptive statistics were used to describe characteristics of study participants, which were then summarised and presented in text and tables. Categorical variables were summarised using frequencies and percentages and the results presented in tables. Continuous variables were summarised using the mean and standard deviation if the data were normally distributed, and for non-normally distributed continuous variables, we used median and interquartile range. The outcome variable was pre-hospital delay, a binary variable which was defined as time from onset of symptoms of stroke to time of arrival at hospital offering essential stroke services of more than 3 h. The prevalence of pre-hospital delay among acute stroke patients was computed by dividing number of acute stroke patients with pre-hospital delay by total number of acute stroke patients in the study.

To determine factors (socio-demographics, symptom onset situational factors, health system factors, and clinical factors) associated with prehospital delay; the study population was divided into participants who had prehospital delay and those who did not have prehospital delay. Since a large prevalence of pre-hospital delay above 15% was obtained and the data was normally distributed, at bivariate analysis, modified Poisson regression was used to test the association between the outcome and each categorized social demographic, symptom onset situational, health system factors and clinical factors. A significant *p*-value was set at the 5% level for all analyses. Prevalence ratio (with 95% confidence interval) was used as the measure of association.

At multivariate analysis, using modified Poisson regression, factors associated with pre-hospital delay at bivariate analysis and key socio-demographics such as gender, and age were included in a multivariable model alongside potential confounders. A *p* value of 0.2 was used as a cut-off to determine variables for inclusion into the modified Poisson regression model to build a final model using backward elimination. Variables with zero counts in some of the cells were not selected despite have significant *p*-values. Confounding and interaction was assessed. A *p* value less than 0.05 and 95% confidence intervals was used as level of significance.

### Ethical considerations

Ethical approval was sought from the School of Medicine Research and Ethics Committee (SOMREC) of Makerere University College of Health Sciences, REC number Mak-SOMREC-2021–227. Administrative clearance to conduct the study at both Mulago and Kiruddu National referral hospitals was sought from the respective institutions. Written informed consent was obtained from all participants or their caregivers to participate in the study. Confidentiality was ensured using code numbers instead of patients’ names. Questionnaires were stored in a locked cabinet for safety.

## Results

### Profile of the study

**Fig. 1:**
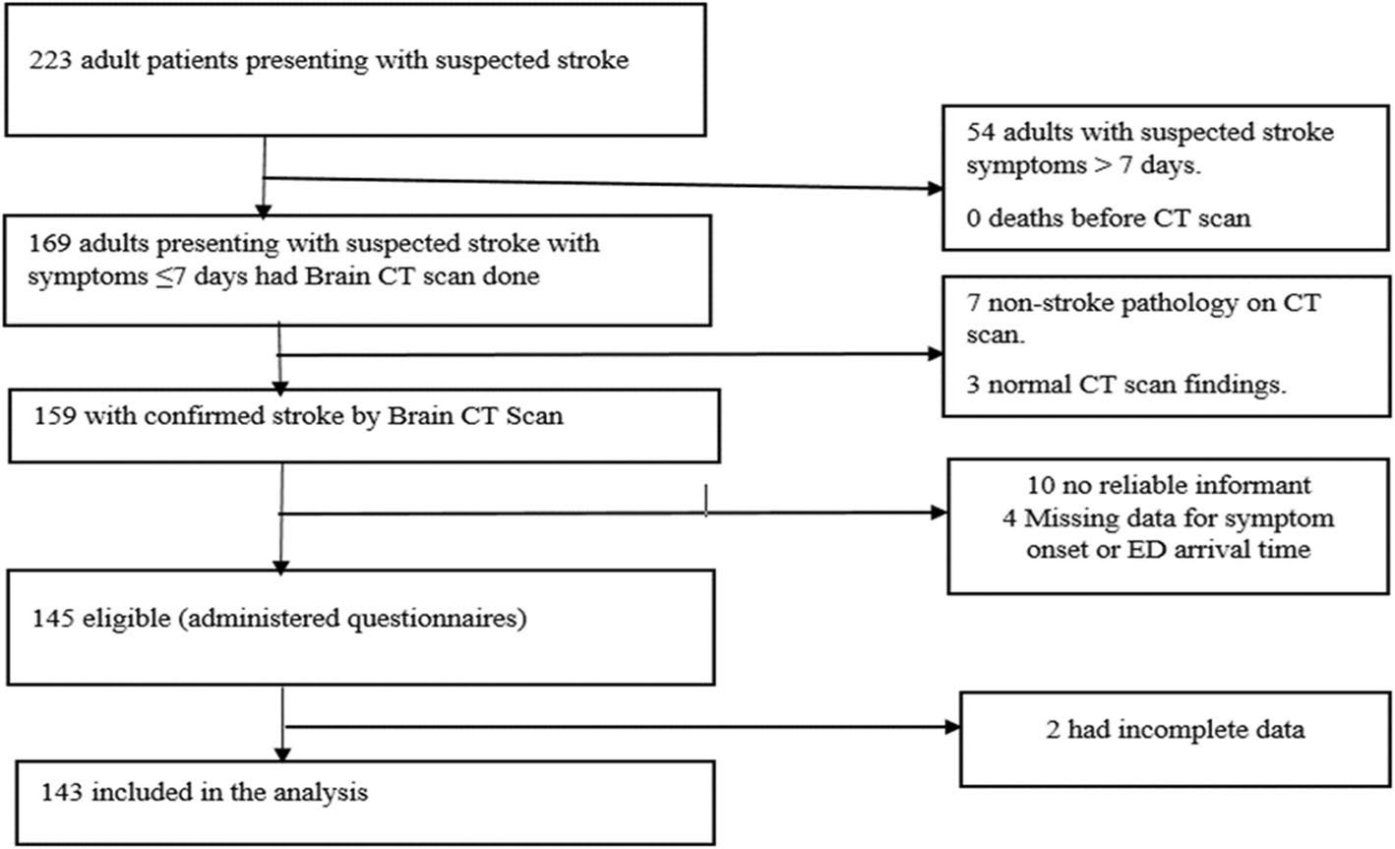
Study flow diagram

The study enrolled 145 acute stroke patients between 2^nd^ February and 30^th^ April 2022 at the Emergency units of Mulago and Kiruddu national referral hospitals. Due to missing data for 2 acute stroke patients, we analysed data for 143 acute stroke patients as illustrated in fig. 1 below.

### Socio-demographic characteristics of the study population

The mean age (SD) of the study participants was 59 (SD = 16) years. The majority of participants were male (51.7%), from urban areas (54%). At least 73% of the participants had attained a primary level of education. Details of the socio-demographic characteristics of the study participants are as shown in Table 1.

**Table 1:** Socio-demographic characteristics of the study participants, n (column %)

	**Mulago Hospital**	**Kiruddu Hospital**	**Total**
	*n* = 86 (60%)	57 (40%)	*n* = 143
**Age categories**			
< 30 years	3 (3.5)	4 (7)	7 (4.9)
30–49 years	24 (27.9)	8 (14)	32 (22.4)
50–59 years	23 (26.7)	7 (12.3)	30 (21)
60–69 years	14 (16.3)	14 (24.6)	28 (19.6)
70 years and above	22 (25.6)	24 (42.1)	46 (32.2)
**Sex (Female)**	46 (53.5)	23 (40.4)	69 (48.3)
**District**			
Kampala	22 (25.6)	9 (15.8)	31 (21.7)
Wakiso	13 (15.1)	23 (40.4)	36 (25.2)
Mukono	12 (14)	2 (3.5)	14 (9.8)
Other	39 (45.3)	23 (40.4)	62 (43.4)
**Highest level of education**			
Tertiary	11 (12.8)	9 (15.8)	20 (14)
Secondary	28 (32.6)	6 (10.5)	34 (23.8)
Primary	28 (32.6)	22 (38.6)	50 (35)
Other	19 (22.1)	20 (35.1)	39 (27.3)
**Occupation**			
Employed	80 (93)	51 (89.5)	131 (91.6)
Unemployed	6 (7)	6 (10.5)	12 (8.4)
**Living alone (Yes)**	6 (7)	11 (19.3)	17 (11.9)

### Symptom onset situational factors and health system factors of study participants

Only 13/143 (9%) of acute stroke patients arrived at the tertiary facility within 3 hours (h) from symptom onset, 24/143 (16.8%) arrived within 6 h and 64/143 (44.8%) arrived within 24 h from onset of stroke symptoms. Majority of the participants, 121/143 (84.6%) reported symptoms occurring at home and 118/143 (82.5%) had a bystander when the symptoms occurred. The majority of patients 124/143 (87.4%) visited lower-level facilities prior to referral to the tertiary facility.

Table 2 below summarizes the participants’ decisions and interactions with the health system from onset of stroke symptoms to arrival at the tertiary facility.

**Table 2:** Symptom onset situational factors and health system factors of study participants

	**Mulago NR Hospital**	**Kiruddu NR Hospital**	**Total**
	n/N ( column %)	n/N (column %)	n/N (column %)
**Time from symptom onset to arrival at hospital**			
0 to 3 h	7/86 (8.1)	6/57 (10.5)	13/143 (9.1)
3 to 6 h	9/86 (10.5)	2/57 (3.5)	11/143 (7.7)
6 to 12 h	12/86 (14)	7/57 (12.3)	19/143 (13.3)
12 to 24 h	11/86 (12.8)	10/57 (17.5)	21/143 (14.7)
> 24 h	47/86 (54.7)	32/57 (56.1)	79/143 (55.2)
**Location when symptom occurred**			
Home	72/86 (83.7)	49/57 (86)	121/143(84.6)
Work	7/86 (8.1)	3/57 (5.3)	10/143 (7)
In transit	3/86 (3.5)	1/57 (1.8)	4/143 (2.8)
Other	4/86 (4.7)	4/57 (7)	8/143 (5.6)
**Presence of bystander at symptom onset(Yes)**	78/86 (90.7)	40/57 (70.2)	118/143 (82.5)
**First decision taken after stroke onset**			
Go to health facility	54/86 (62.8)	15/57 (26.3)	69/143 (48.3)
Ask family member or relative to help	5/86 (5.8)	18/57 (31.6)	23/143 (16.1)
Ask friend or neighbours to help	2/86(2.3)	10/57 (17.5)	12/143 (8.4)
Seek spiritual healing (prayers)	9/86 (10.5)	1/57 (1.8)	10/143 (7)
Called family doctor	3/86 (3.5)	5/57 (8.8)	8/143 (5.6)
Others	13/86 (15.1)	8/57 (14)	21/143 (14.7)
**Had money at home at symptom onset**			
Yes	36/86 (41.9)	27/57 (47.4)	63/143 (44.1)
No	50/86 (58.1)	30/57 (52.6)	80/143 (55.9)
**Means of transport to tertiary facility**			
Owned	20/86 (23.3)	15/57 (26.3)	35/143 (24.5)
Hired	40/86 (46.5)	27/57 (47.4)	67/143 (46.9)
Government ambulance	26/86 (30.2)	15/57 (26.3)	41/143 (28.7)
**Came directly to tertiary hospital**			
Yes	15/86 (17.4)	3/57 (5.3)	18/143(12.6)
No	71/86 (82.6)	54/57 (94.7)	125/143 (87.4
**Level of health facility first visited ( *N* = 125)**			
Private clinic	24/71 (33.8)	26/54 (48.1)	50/125 (40)
Private hospital	13/71 (18.3)	7/54 (13)	20/125 (16)
HC IV	24/71 (33.8)	12/54 (22.2)	36/125 (28.8)
HC III	4/71 (5.6)	5/54 (9.3)	9/125 (7.2)
Other	6/71 (8.5)	4/54 (7.4)	10/125 (8)

*HC IV* Health centre IV

### Clinical characteristics of study participants

At admission to the tertiary facility, 94/143 (65.7%) had ischemic stroke, 33/143 (23%) were unconscious with a GCS ≤ 8/15 and 129/143 (90.2%) had asymmetrical arm weakness. The clinical characteristics of the study participants are as detailed in Table 3.

**Table 3:** Clinical characteristics of study participants

	**Frequency *n* = 143**	**Percent**
*Past medical history*		
**History of stroke or TIA**		
Yes	28	19.6
No	115	80.4
**Family member or acquaintance with history of stroke or TIA**		
Yes	20	14
No	123	86
**History of Hypertension**		
Yes	97	67.8
No	46	32.2
**History of Diabetes Mellitus**		
Yes	31	21.7
No	112	79.3
**History of HIV**		
Yes	19	13.3
**Was attending a chronic care clinic**		
Yes	64	44.8
No	79	55.2
**Reported current tobacco use (smoking)**		
Yes	12	8.4
No	131	91.6
**Reported regular intake of alcohol**		
Yes	40	28
No	103	72
*Clinical status at admission to tertiary facility*		
**Stroke subtype**		
Ischemic stroke	94	65.7
Haemorrhagic stroke	49	34.3
**Glasgow coma scale score at admission**		
GCS 9–15	110	76.9
GCS ≤ 8	33	23.1
**Motor aphasia**		
Yes	99	69.2
No	44	30.8
**Asymmetrical facial weakness**		
Yes	104	72.7
No	39	27.3
**Asymmetrical arm weakness**		
Yes	129	90.2
No	14	9.8
**Asymmetrical leg weakness**		
Yes	128	89.5
No	15	10.5

### Prevalence of pre-hospital delay among adult acute stroke patients at Mulago and Kiruddu hospitals in Kampala

The study found a prevalence of pre-hospital delay of 90.9% (131/143); (95% confidence interval (CI): 84.9%—94.7%) among study participants.

### Factors associated with pre-hospital delay among adult acute stroke patients presenting to Mulago and Kiruddu National Referral hospitals in Kampala

#### Socio-demographic factors associated with prehospital delay

At bivariate analysis, participants from areas outside Kampala district (Wakiso district and beyond) had a significantly higher prevalence of pre-hospital delay of 98.7% compared to people from Kampala district 74.2%, *p* = 0.008. Participants who were living alone had a significantly higher prevalence of prehospital delay of 100% than those who lived with somebody (relatives, friends, etc.), 89.7%, *p* < 0.001. There was no significant difference between the prevalence of prehospital delay among participants at both Mulago and Kiruddu national referral hospitals. Prevalence of pre-hospital delay did not significantly vary across age and sex. Table 4 shows the association between prehospital delay and socio-demographic factors at bivariate analysis.

**Table 4:** Socio-demographic factors associated with pre-hospital delay

	**Timely visit**	**Pre-hospital delay**				
	***n* = 13 (%)**	***n* = 130 (%)**	**Crude PR (95% CI)**	***P* value**	**Adjusted PR (95% CI)**	***P* value**
**Study site**						
Mulago NR Hospital	7 (8.1)	79 (91.9)	1		1	
Kiruddu NR Hospital	6 (10.5)	51 (89.5)	0.97 (0.87–1.09)	0.637	0.98 (0.96 -1.00)	0.013
**Age**						
< 50 years	5 (12.8)	34 (87.2)	1			
50-69 years	3 (5.2)	55 (94.8)	1.09 (0.95–1.24)	0.222		
70 + years	5 (10.9)	41 (89.1)	1.02 (0.87–1.20)	0.783		
**Sex**						
Female	9 (13)	60 (87)	1			
Male	4 (5.4)	70 (94.6)	1.09 (0.98–1.21)	0.122		
**District**						
Kampala	8 (25.8)	23 (74.2)	1		1	
Wakiso	4 (11.1)	32 (88.9)	1.2 (0.94–1.52)	0.137	1.19 (1.06–1.33)	0.003
Other (> 25 km from hospital)	1 (1.3)	75 (98.7)	1.33 (1.08–1.64)	0.008	1.28 (1.22–1.34)	< 0.001
**Highest level of education**						
Tertiary	2 (10)	18 (90)	1			
Secondary	6 (17.6)	28 (82.4)	0.92 (0.74–1.13)	0.416		
Primary	1 (2)	49 (98)	1.09 (0.94–1.27)	0.272		
No education / not known	4 (10.3)	35 (89.7)	1 (0.83–1.20)	0.975		
**Occupation**						
Employed	13 (9.9)	118 (90.1)	1			
Unemployed	0 (0)	12 (100)	1.11 (1.05–1.18)	< 0.001		
**Living alone**						
No	13(10.3)	113 (89.7)	1			
Yes	0 (0)	17 (100)	1.12 (1.05–1.18)	< 0.001		

Footnote: PR –prevalence ratio

At multivariate analysis, participants from Wakiso and other parts of Uganda had a prevalence of prehospital delay that was 19% and 28% higher than that of participants from Kampala, PR: 1.19 (1.06—1.33) and PR: 1.28 (1.22—1.34) respectively.

#### Symptom onset situational factors and health system factors associated with pre-hospital delay

At bivariate analysis, participants who went direct to the tertiary hospital had a significantly lower prevalence (61.1%) of prehospital delay than those who did not, 95.2%, *p* = 0.001. Participants who got symptoms at work were significantly delayed compared to those who got symptoms at home, *p* < 0.001. Presence of bystander did not predict prehospital delay. Table 5 shows association of prehospital delay and participants’ decisions and interaction with the health system from onset of stroke symptoms to arrival at the tertiary facility.

**Table 5:** Symptom onset situational factors and health system factors associated with pre-hospital delay

	**Prehospital delay**					
	**No**	**Yes**	**Crude PR** **(95% CI)**	***p* value**	**Adjusted PR (95% CI)**	***p* value**
	**n (row%)**	**n (row%)**				
**Location when symptoms occurred (*N* = 143)**						
Home	12 (9.9)	109 (90.1)	1			
Work	0 (0)	10 (100)	1.11 (1.07–1.16)	< 0.001		
In transit	1 (25)	3 (75)	0.83 (0.58–1.20)	0.329		
Other	0 (0)	8 (100)	1.11 (1.07–1.16)	< 0.001		
**Bystander present at symptom onset (*N* = 143)**						
No	3 (12)	22 (88)	1			
Yes	10 (8.5)	108 (91.5)	1.04 (0.86–1.25)	0.678		
**First decision taken stroke onset (*N* = 143)**						
Go to health facility	8(11.6)	61 (88.4)	1			
Ask family or relative for help	3 (13)	20 (87)	0.98 (0.85–1.14)	0.828		
Ask friend or neighbour to help	0(0)	12 (100)	1.13 (1.04–1.23)	0.003		
Seek spiritual healing	0 (0)	10 (100)	1.13 (1.04–1.23)	0.003		
Called family doctor	1(12.5)	7 (87.5)	0.99 (0.87–1.13)	0.876		
Others	1 (4.8)	20 (95.2)	1.08 (0.92–1.26)	0.349		
**Had money at home at symptom onset (*N* = 143)**						
No	4 (5)	76 (95)	1			
Yes	9 (14.3)	54 (85.7)	0.9 (0.78 -1.04)	0.149		
**Means of transport to tertiary hospital (*N* = 143)**						
Owned	8 (22.9)	27 (77.1)	1		1	
Hired/Government ambulance	5 (4.6)	103 (95.4)	1.24 (1.12–1.37)	0.004	1.17 (1.13–1.20)	< 0.001
**Came directly to tertiary Hospital (*N* = 143)**						
No	6(4.8)	119 (95.2)	1			
Yes	7(38.9)	11 (61.1)	0.64 (0.50–0.82)	0.001		
**Level of health facility first visited (*N* = 125)**						
Private clinic	5 (10)	45 (90)	1			
Private hospital	0 (0)	20 (100)	1.11 (1.07–1.15)	< 0.001		
HC IV	1 (2.8)	35 (97.2)	1.08 (1.04–1.12)	< 0.001		
HC III	0 (0)	9 (100)	1.11 (1.07–1.15)	< 0.001		
Other	0 (0)	10 (100)	1.11 (1.07–1.15)	< 0.001		

*PR* Prevalence ratio

At multivariable analysis, the prevalence of prehospital delay among participants who did not own their own means of transport was 17% higher than those who owned their means of transport PR: 1.17 (1.13—1.20).

### Clinical characteristics associated with pre-hospital delay

At bivariate analysis, the prevalence of prehospital delay was lower among participants with history of hypertension and participants in chronic care. Having a history of stroke was not associated with prehospital delay. The subtype of stroke and the GCS level were not associated with prehospital delay. None of the clinical factors was significant at multivariate analysis. Table 6 below summarizes the bivariate analysis of clinical characteristics associated with prehospital delay.

**Table 6:** Clinical characteristics associated with pre-hospital delay

	**Prehospital delay**					
	**No** **(*N* = 13)**	**Yes** **(*N* = 130)**	**Crude PR** **(95% CI)**	***p* value**	**Adjusted PR (95% CI)**	***p* value**
	**n ( row %)**	**n (row %)**				
**History of stroke or TIA**						
No	11 (9.6)	104 (90.4)	1			
Yes	2 (7.1)	26 (92.9)	1.03 (0.80–1.32)	0.836		
**Stroke or TIA history (Family member/friend)**						
No	12 (9.8)	111 (90.2)	1			
Yes	1 (5)	19 (95)	1.05 (0.96–1.15)	0.255		
**History of Hypertension**						
No	3(6.5)	43(93.5)	1			
Yes	10 (10.3)	87 (89.7)	0.96 (0.95–0.97)	< 0.001		
**History of Diabetes Mellitus**						
No	10 (8)	115(92)	1			
Yes	3 (9.7)	28 (90.3)	0.99 (0.92–1.07)	0.834		
**History of HIV**						
No	13 (10.5)	111(89.5)	1			
Yes	0 (0)	19 (100)	1.12 (1.10–1.14)	< 0.001		
**Attending a chronic care clinic**						
No	6 (7.6)	73 (92.4)	1			
Yes	7 (10.9)	57 (89.1)	0.96 (0.96–0.96)	< 0.001		
**Current tobacco use**						
No	13 (9.9)	118 (90.1)	1			
Yes	0 (0)	12 (100)	1.11 (1.08–1.14)	< 0.001		
**Regular use of alcohol**						
No	10 (9.7)	93 (90.3)	1			
Yes	3 (7.5)	37 (92.5)	1.02 (0.97–1.08)	0.363		
**Stroke subtype at admissi**						
Ischemic stroke	8(8.6)	86 (91.4)	1			
Haemorrhagic stroke	5 (10.2)	44 (89.8)	0.97 (0.81–1.17)	0.76		
**Glasgow Coma Scale at admission**						
GCS 9–15	9 (8.2)	101 (91.8)	1			
GCS ≥ 8	4 (12.1)	29 (87.9)	1 (0.93–1.09)	0.928		
**Aphasia**						
Absent	6 (13.6)	38 (86.4)	1			
Present	7 (7.1)	92 (92.9)	1.08 (0.77–1.51)	0.673		
**Asymmetrical facial weakness**						
Absent	7 (17.9)	32 (82.1)	1			
Present	6 (5.8)	98 (94.2)	1.15 (0.98–1.34)	0.081		
**Asymmetrical arm weakness**						
Absent	3 (21.4)	11 (78.6)	1			
Present	10 (7.8)	119 (92.2)	1.17 (0.91–1.51)	0.214		
**Asymmetrical leg weakness**						
Absent	3 (20)	12 (80)	1			
Present	10 (7.8)	118 (92.2)	1.15 (0.95–1.40)	0.15		

*PR* prevalence ratio

## Discussion

### Prevalence of pre-hospital delay among acute stroke patients attending Kiruddu and Mulago tertiary Hospitals

The study assessed the prevalence of prehospital delay among acute stroke patients. We found a very high prevalence of pre-hospital delay of 90.9% similar to studies carried out elsewhere in tertiary hospitals in Africa such as Nigeria that reported prevalence of 89.2% and Egypt that found 100% prevalence of pre-hospital delay [11, 14]. Another recent South African study of 730 patients with stroke, only 13% presented within 4.5 h of symptom onset and the median time from onset of symptoms to presentation to hospital was 24 h [15]. Similarly, in other low and middle-income countries, a high prevalence of prehospital delay has been reported in multiple studies. Several studies from Asia among patients with acute stroke found prehospital delay prevalence of 69.3%, 85.7%, and 79.8 [16–18]. However, some of these studies defined pre-hospital delay differently, with a cut-off of > 4 h. This implies that pre hospital delay is still a major challenge in management of stroke worldwide.

Studies from some high-income countries (HIC) in regions of Europe and Asia showed lower prevalence of prehospital delay. In these studies, prevalence of less than 50% for pre hospital delay among acute stroke patients was observed [19, 20]. Hence, acute stroke patients in low and middle countries generally present much later than patients in high-income countries. This could be explained by low community awareness of relevance of early arrival to facility that can offer essential stroke services and poorly developed stroke pre-hospital referral systems.

### Factors associated with pre-hospital delay among adult acute stroke patients

Patients that stayed outside Kampala district were more likely to have pre-hospital delay as compared to those who stayed within Kampala district. This is similar to other studies that found increased distance away from the tertiary hospital as a predictor of pre hospital delay [13, 17, 21]. Anecdotally, inefficient road systems including traffic jam could contribute to delays for stroke patients travelling from beyond Kampala district.

Patients who used hired or government cars had a higher prevalence of pre-hospital delay as compared to those who used personal vehicles. Contrary to this, other studies found that using an EMS ambulance was associated with less pre-hospital delay [13, 22, 23]. In addition, O’Meara et al. found that majority of the prehospital delays occur prior to calling EMS services [15]. In this study, its postulated that patients who used government ambulances could have delayed due to delays in getting authorisation from the hospital administrator or patient attendants mobilising money to fuel the ambulances.

According to the study, at bivariate analysis, living alone was associated with pre-hospital delay much as the association was not significant at multivariate analysis. This is similar to another study that demonstrated that living alone increased chances of pre-hospital delay among patients with acute stroke as compared to those who stayed with someone [24]. It is likely that at onset of stroke symptoms, people living alone may take longer to find someone to help them compared with those living with someone.

Again, at bivariate analysis, patients or their proxy whose first decision was to call a friend or neighbour and those that sought spiritual healing first were associated with a delay as compared to those who went direct to hospital. This was however not significant at multivariate analysis probably due to small sample size. This is comparable to another study by Moser et al. that found higher chances of pre-hospital delay among patients that called a friend or relative as a first decision [24]. Patients who called a general practitioner or family doctor were associated with less pre-hospital delay. These findings are similar to a study done in Switzerland that found less chances of pre hospital delay among patients that first called a doctor [19]. However, the same study also found out that half of the patients who first called a doctor had a face-to-face visit to the family doctor before hospital admission, which quadrupled the odds of prehospital delay [19].

Furthermore, patients who came directly to the tertiary hospital had less pre-hospital delay as compared to those who went to a lower-level facility or private facility at bivariate analysis. This is also supported by other studies that found coming directly to tertiary hospital associated with less pre hospital delay [13, 18, 21]. Iyer et al. found that patients who visited a nearby clinic before reaching arriving at the hospital were significantly delayed [18]. Another study conducted in Nepal also found the odds of being early to be over three times more in patients who presented directly to the emergency department [17].

Patients who initially visited a public health facility had higher chances of prehospital delay as compared to those who visited a private clinic. These findings are similar to studies conducted in Indonesia, and Egypt that found an association between patients referred from public facilities and pre-hospital delay [14, 25].

Presence of a bystander was not associated with prehospital delay in this study despite more patients having a bystander at the time of onset of a stroke in this study. This is contrary to a study by Reeves et al. found that having a bystander at onset of a stroke substantially reduced pre-hospital delay [26]. A knowledgeable bystander would be expected to call for appropriate help as soon as possible. Therefore, knowledge of the bystander likely is an intermediary variable between the association of bystander and prehospital delay.

Level of consciousness, aphasia and asymmetrical arm weakness were all associated with higher likelihood of pre-hospital delay. This is contrary to some other studies, which have shown that patients with severe symptoms had lower odds of pre hospital delay [27, 28]. Some of the other studies have demonstrated that patients with speech disturbances had higher chances of coming early to hospital [18, 29]. Patients with more severe symptoms could have delayed at peripheral facilities receiving treatment to stabilise them.

Patients with hypertension, diabetes as well as those attending a chronic care clinic were less likely to have pre-hospital delay. Patients in a chronic care clinic perhaps receive health education which possibly improves their ability to recognize stroke symptoms. However, other studies found out that patients with comorbidities such as hypertension, and diabetes were not significantly better in seeking timely care after a stroke [16, 20]. Having a history of stroke was not associated with pre-hospital delay. This is contrary to a study from urban China that found that patients with a history of stroke had higher odds of pre-hospital delay [16]. Another study however reported lower odds of pre-hospital delay among patients with history of stroke [23]. It would be expected that stroke survivors would have more awareness and capacity to act to reduce stroke related pre-hospital delay.

### Strength and weakness

This study provides useful information for addressing pre-hospital delay among acute stroke patients presenting to facilities, which can offer key essential stroke services, in similar resource-limited settings. Patients were recruited from the two main national referral hospitals; stroke was confirmed by brain CT scan and information was collected in real time as patients arrived at the emergency wards. Larger multicentre studies are needed in large cohort of patients involving different stroke centres to confirm our findings.

We acknowledge the following limitations in our study. The study sample size was adjusted for only 5% which resulted in a small sample size that could have affected the power of the study, which resulted in less variables being assessed at the multivariable level. Nevertheless, findings from the study lay the foundation for future studies on delays related to stroke care in similar settings. The study participants might have had recall bias for time of onset of symptoms provided by stroke patients or their attendants. This was minimised by choosing the most reliable informant. We excluded a few unconscious patients who did not have a reliable informant so possible selection bias, yet they could have presented early since the very ill may present quicker. This could possibly overestimate the prevalence of pre-hospital delay. Also, this was a cross sectional study so we could not determine causality between the determined factors and prehospital delay. Furthermore, the study did not explore the timeline from symptom onset to arrival to determine which phase contributes most to the prehospital delay. In addition, factors contributing to prehospital delay among stroke patients could have been further explored qualitatively.

## Conclusions and recommendations

The prevalence of hospital delay among acute stroke patients presenting to public tertiary hospitals in Uganda is very high. Staying outside the Kampala district and using hired or government ambulance for transport to tertiary facility were associated with prehospital delay. Interventions aimed at promoting early arrival of acute stroke patients to tertiary facilities which can offer essential stroke services should be implemented. The capacity of health facilities outside Kampala should be enhanced to offer essential stroke services to manage acute stroke patients. Efforts to reduce pre-hospital delay should include improving pre-hospital transport systems to expedite transfer of acute stroke patients to appropriate tertiary facilities.

## Data Availability

The datasets used during this study are available from the corresponding author on request.
